# JAK2V617F Allele Burden Measurement in Peripheral Blood of Iranian Patients with Myeloproliferative Neoplasms and Effect of Hydroxyurea on JAK2V617F Allele Burden

**Published:** 2016-04-01

**Authors:** Shirin Ferdowsi, Seyed H. Ghaffari, Naser Amirizadeh, Azita Azarkeivan, Kamran Atarodi, Mohammad Faranoush, Gholamreza Toogeh, Reza Shirkoohi, Mohammad Vaezi, Mahtab Maghsoodlu, Kamran Alimoghaddam, Ardeshir Ghavamzadeh, Hosein Teimori Naghadeh

**Affiliations:** 1 Blood Transfusion Research Center, High Institute for Research and Education in Transfusion Medicine, Tehran, Iran; 2 Hematology-Oncology and Stem Cell Transplantation Research Center, Tehran University of Medical Sciences, Tehran, Iran; 3 Hematology-Oncology and BMT Research Center, Imam Khomeini Hospital, Tehran University of Medical Sciences, Tehran, Iran; 4 Molecular Genetics, Cancer Research Center, Cancer Institute, Imam Khomeini Hospital Complex, Tehran University of Medical Sciences, Tehran, Iran

**Keywords:** Hydroxyurea, JAK2V617F, Myeloproliferative neoplasms

## Abstract

**Background:** Myeloproliferative neoplasms (MPNs) are clonal malignant diseases that represent a group of conditions including polycythemia vera (PV), essential thrombocythemia (ET) and primary myelofibrosis (PMF). The aim of this study was to evaluate possible correlations between JAK2V617F allele burden and clinicohematologic characteristics in Iranian patients with MPNs. We also aimed at determining the correlation between JAK2V617F allele burden and use of cyto reductive treatment (hydroxyurea).

**Materials and Methods:** We performed ARMS-PCR for all MPNs samples and subsequently performed real-time quantitative polymerase chain reaction (qRT-PCR) for JAK2V617F allele burden measurement using DNA from peripheral blood leukocytes.

**Results:** Two distinct groups of patients were examined at a single time point: group A (n=40; 20 PV, 20 ET) was examined at the time of diagnosis; group B (n=85; 40 PV, 30 ET and 15 PMF) while under treatment with hydroxyurea (HU). The median allele burden of the JAK2 V617F was 72% for PV and 49% for ET patients at the time of diagnosis (p=0.01). For patients with HU treatment, we determined the median JAK2V617F allele burden to be 43%, 40%, and 46.5 % in PV, ET and PMF patients; respectively. HU-treated PV patients had a significant lower %JAK2V617F than PV patients at the time of diagnosis (43% vs. 72%, p=0.005). In ET group, the relationship between the JAK2 V617F allele burden and leukocyte count was significant (p=0.02 and p=0.01 in untreated and treated patients, respectively).

**Conclusions:** Our results showed that patients with PV have a higher JAK2V617F allele burden. Moreover, our study demonstrated that the JAK2V617F allele burden correlates with clinical features in ET group. We also showed hydroxyurea can affect the JAK2V617F allele burden in PV patients.

## Introduction

The JAK2V617F mutation, which occurs in most patients with polycythemia vera (PV), essential thrombocythemia (ET) and primary myelofibrosis (PMF), is considered integral to the pathogenesis of myeloproliferative neoplasms (MPNs). ^1^^-^^3^  There is now a growing interest in the JAK2 V617F allele burden (% JAK2 V617F) and its potential influence on disease phenotype. Several studies have shown a higher burden of the JAK2V617F allele in PV than in the ET. ^4^^-^^7^  Limited studies are available from Asian populations. ^5^^,^^8^^-^^10^  On the other hand, hydroxyurea (HU) is widely used as a first line myelosuppressive therapy in these patients^11^ but the effect upon the JAK2V617F allele burden is still controversial. ^12^^-^^17^  Therefore, in this study, we employed quantitative assay for V617F allele in a series of MPNs patients, with the aim to determine how the JAK2V617F allele burden correlated with laboratory and clinical features of the disease. We also aimed at determining the correlation between JAK2V617F allele burden and use of cytoreductive (HU) drug. To our knowledge, this report is the first of its kind from Iran.

## MATERIALS AND METHODS

**Patients and samples:** Blood samples were obtained from patients (n=125) with PV, ET and PMF between 2007 and 2014. The original diagnosis criteria were established by Polycythemia Vera Study Group (PVSG).^   18 ^ Two distinct groups of patients were examined at a single time point: group A (n=40; 20 PV, 20 ET) at the time of diagnosis and group B (n=85; 40 PV, 30 ET and 15 PMF) during HU therapy. The control group consisted of 20 healthy subjects. The patients were selected from Hematology-Oncology and BMT Research Center at Shariati and Imam Khomeini Hospital affiliated with Tehran University of Medical Science. The study was approved by our institutional review board and written informed consent was obtained from all patients. (Ethical code: ir.tums.horcsct.1394.103.7)

**JAK2 V617F screening by amplification refractory mutation system-polymerase chain reaction (ARMS-PCR)**

**Figure 1:**
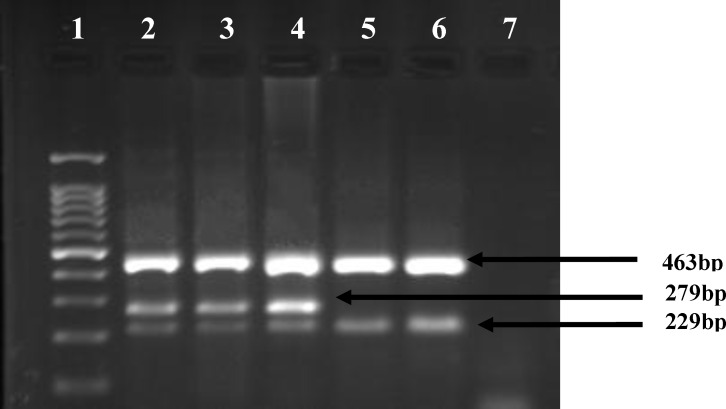
Agarose gel analysis for the detection of JAK2V617F mutation in genomic DNA by ARMS- PCR. The 463- bp fragment was amplified as a control band for all PCR products. wild-type specific primers produce a fragment of 229- bp and mutant specific primers generate a fragment of 279-bp. Lane 2, 3 and 4 samples from patients with mutation; lane 5 sample from patients without mutation; lane 6 is a healthy person, lane 7 is negative PCR control. Lane 1 is 100-base pair (bp).

Genomic DNA was prepared from leukocytes using the DNA blood mini kit (Qiagen, Germany). Mutation analysis of the JAK2 V617F was initially performed using ARMS-PCR.^   19 ^ PCR primers were Forward Outer (FO): 5’- TCCTCAGAACGT TGA TGGCAG-3’, Reverse Outer (RO): 5’-ATTGCTTTCCTTTTTCACAAGAT-3’, forward wild-type specific (FWt): 5’- GCATTTGGT TTTAAATTATGGAGTATATG -3’ and Reverse mutant-specific (RMt): 5’- GTTTTACTTACTCTCGTCTCCACAAAA-3’. The FO and RO primers generate a control 463-bp band in all cases. The Rmt and the FO primers generate a 279-bp mutant. In the presence of wild-type JAK2 the RO and the Fwt primers produce a fragment of 229-bp. The PCR reaction was performed in a total volume of 25 μL containing approximately 50 ng DNA, 12.5 μL of PCR Master Mix 2X (Roche, Germany), 0.5 μL of each FO ، RO and Fwt, and 1μL of Rmt primer. The PCR conditions on the thermal cycler (Eppendorf) were as follows: denaturation at 94°C for 6 minutes, followed by 40 cycles of 40 sec at 94°C, 45 sec at 56°C, 45 sec at 72°C, and the final extension step of 10 min at 72°C. A total of 10 μL from the PCR product were electrophoresed on 3% standard agarose gels (Sigma, Germany) at 80 V for 25 min. The fragments were visualized by ethidium bromide under UV transilluminator (Figure 1).

**Quantification of JAK2 V617F mutation by real- time quantitative polymerase chain reaction (qRT-PCR)**

**Figure 2:**
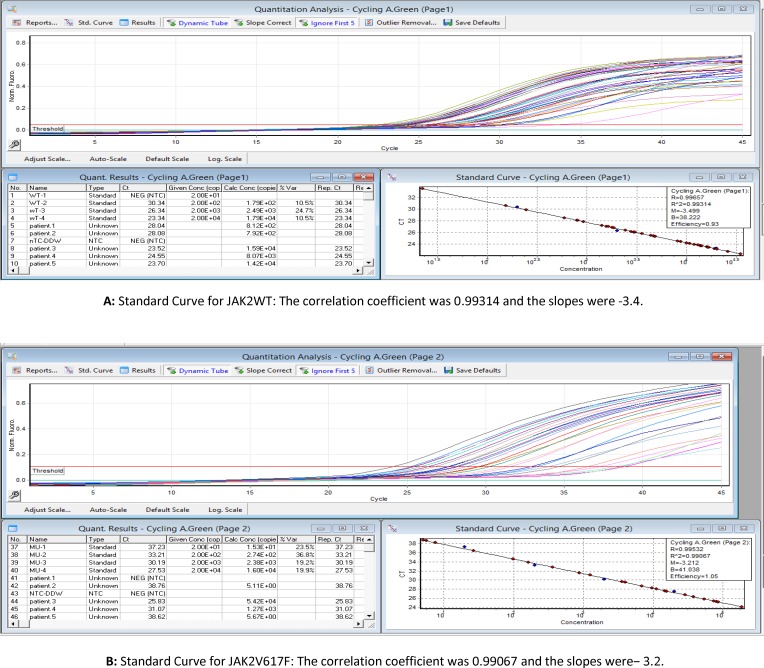
Standard curves for real-time PCR (A: Results for JAK2WT, B: Results for JAK2V617F

For quantitative analysis of the JAK2 V617F mutation, we performed qRT-PCR using JAK2 AB analitica kit (RS-JAK2V617FQ-Italy) according to the Manufacturer’s instructions. Briefly, five μL of genomic DNA was added to 20 μL of the RQ-PCR premix solution (V617F or wild type) in each well. A 45-cycle PCR was performed on a Rotor-Gene 6000 real-time analyzer (Qiagen) according to the following cycling conditions: 10 minutes at 95°C followed by 45 cycles of 15 seconds at 95°C and 1 min at 60°C. Standard curves for both V617F and wild type were constructed using either a V617F or wild-type standards with known concentrations (20, 200, 2000, 20000 DNA copies/µl) provided by the manufacturer. The equation was calculated for each curve, and these equations were used to calculate the copy number of V617F and wild-type alleles in unknown samples. The percentage of JAK2V617F was calculated using the DNA copy numbers according to the following formula: JAK2V617F/ (JAK2V617F + JAK2WT) × 100 (Figure 2).

**Statistical analysis**

The nonparametric Mann–Whitney test was used to compare JAK2V617F allele burden between categories of MPNs. Correlations between JAK2V617F allele burden and laboratory parameters (white blood cell count, hemoglobin value, and platelet count) were determined using Spearman’s rank correlation test. The statistical significance level was set at 5% (p ≤ 0.05).

## Results

**Figure 3:**
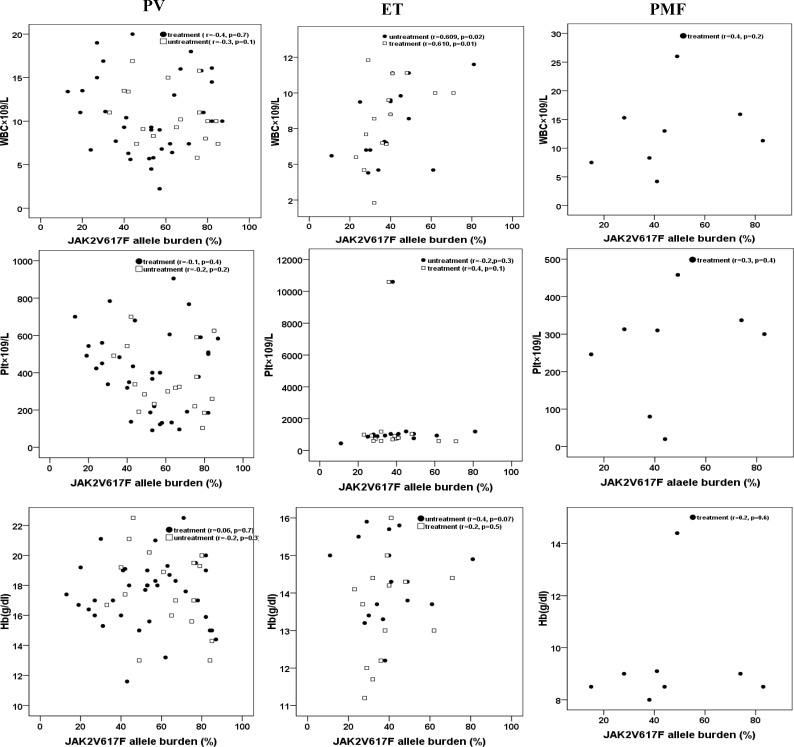
Correlation between JAK2V617F allele burden and hematologic parameters. White blood cell count (WBC), hemoglobin value (Hb) and platelet count (Plt) for JAK2V617F-positive PV (n = 48), ET (n = 30) and PMF (n = 8) patients are presented.

 To examine the JAK2V617F mutation and determine the allele burden of the mutant gene, we performed ARMS-PCR for all MPNs samples and subsequently performed qRT-PCR for positive JAK2 mutation samples. In total, 80% of PV patients (48/60), 63% of ET patients (30/50), and 53.3% of PMF patients (8/15) were positive for JAK2V617F. All patients were heterozygous for mutation in JAK2V617F using ARMS-PCR method. Forty patients with a clinical diagnosis of PV and ET were examined at the time of diagnosis. Eighty- five patients (40 PV, 30 ET and 15 PMF) were examined during HU therapy. The median duration of treatment was 2 years (Table 1 and 2). The median allele burden of the JAK2V617F was 72% (68.07 ± 16.7) for PV and 49% (51.4 ± 17.3) for ET patients at the time of diagnosis (p=0.01). For patients with HU treatment, we determined the median JAK2V617F allele burden to be 43% (44.8 ± 22.2), 40% (35.4 ± 11.7) and 46.5% (48.8 ± 26.08) in PV, ET, and PMF patients, respectively. There was no difference in % JAK2V617F between ET patients under treatment and those tested at the time of diagnosis (40% vs. 49%, p=0.1). In contrast, HU-treated PV patients had lower % JAK2V617F than untreated patients at the time of diagnosis (43% vs. 72%, p=0.005). The analysis of PV showed that the decrease in % JAK2V617F caused by HU therapy was significant both in women (48% with HU vs. 82% at diagnosis, p=0.03) and men (25% with HU vs. 58% at diagnosis, p=0.02). The additional analysis showed no statistically significant relationship between the JAK2 V617F allele burden and leukocyte, hemoglobin, or platelet counts in PV and PMF. But the relationship between the JAK2 V617F allele burden and leukocyte count was significant (p=0.02 and p=0.01 in untreated and treated patients, respectively) in ET group (Figure 3). Patients were categorized according to JAK2 V617F allele frequency into two groups: those with a low allele burden (patients with a mutation frequency <50%; n=50) and those with a high allele burden (patients with a mutation frequency ≥50%; n=36). Patients with ≥50% mutational load were slightly older than those with <50% mutational load (mean age: 53 yr vs. 44 yr). The mean hemoglobin level was higher in patients with ≥50% mutational load (18.3 g/dl vs. 16.8 g/dl, p=0.04) and mean platelet count was lower in patients with ≥50% mutational load (355×10^9^/L vs. 514×10^9^/L, p=0.04). No significant differences were detected with regard to the other clinical and hematologic parameters in patients with ≥ 50% allele burden.

## Discussion

 To date, the detection and quantification of the JAK2WT and JAK2V617F alleles are usually evaluated using genomic DNA. However, the quantification of JAK2V617F mRNA transcript levels by a qRT-PCR may provide some advantages over the DNA allele burden.^   6 ^ Having compared DNA and RNA samples, Fantasia et al.^   20 ^ found that the ratio of JAK2V617F to JAK2WT was significantly higher at the RNA level, both in PV (p =0.005) and ET (p =0.001) samples, but in a study by Vannucchi et al.^   21 ^ no differences were observed with respect to the use of RNA or DNA on the determination of the JAK2V617F burden. In the present study, the results of using DNA from peripheral blood leukocytes showed that the frequency of the JAK2 mutation is comparable with other reports (Table 3). ^22^^-^^28^  Homozygous for the V617F mutation occurs in about 25% to 30% of patients with PV and PMF but is rare in patients with ET. ^29^^-^^32^  The use of ARMS-PCR showed that all patients with the JAK2V617F mutation were heterozygote. The different incidences may depend on various factors such as the number of patients and the sensitivity of the method used. When clinicohematologic data were compared between JAK2V617F positive and negative patients in the PMF subgroup, no significant differences were detected. In contrast, PV patients with the JAK2V617F mutation had higher counts of white blood cell (p=0.009) and platelet (p=0.01) in under treatment group.

**Table 1:** Clinical and hematologic characteristics at diagnosis in MPN patients (tested at the time of diagnosis)

**Patients**		**%**	**Male/ female**	**Median age**	**Splenomegaly Normal/ Abnormal**	**WBC × 10** ^9^ **/L (Mean ± SD)**		**Hb(g/dl) (Mean ± SD)**		**Plt×10** ^9^ **/L (Mean ± SD)**		
**PV**	**JAKK2V617**	18 (90%)	8/10	48	6/12	11.05 ± 2.99	P=0.1	17.4 ± 2.74	P=0.1		388.4 ± 184	P=0.6
	**JAK2 Wild-type**	2 (10%)	1/1	30	2/0	8.94 ± 3.79		18.6 ± 2.3			234 ± 17.3	
**ET**	**JAKK2V617**	15 (75%)	6/9	59	0/15	10.1 ± 1.1	P=0.004	14.4 ± 0.98	P=0.01		785 ± 179.8	P=0.3
	**JAK2 Wild-type**	5 (25%)	4/1	55	0/5	6.8 ± 2.7		13.1 ± 1.3			1828 ± 3088	

**Table 2:** Clinical and hematologic characteristics at diagnosis in MPN patients (tested while receiving HU)

**Patients**		**%**	**Male/ female**	**Median age**	**Splenomegaly** **Normal/ Abnormal**	**WBC × 10** ^9^ **/L** **(Mean ± SD)**		**Hb (g/dl)** **(Mean ± SD)**		**Plt × 10** ^9^ **/L** **(Mean ± SD)**	
**PV**	**JAKK2V617**	30 (75%)	16/14	52	20/10	21.9 ± 4.1	P=0.009	17.2 ± 1.9	P=0.08	729 ± 181.8	P=0.01
	**JAK2 Wild-type**	10 (25%)	9/1	41	2/8	7.7 ± 3.07		18.7 ± 2.02		270 ± 103.2	
**ET**	**JAKK2V617**	15 (50%)	6/9	55	2/13	8.9 ± 2.3	P=0.01	14.1 ± 1.8	P=0.03	879 ± 204	P=0.6
	**JAK2 Wild-type**	15 (50%)	6/9	51	1/16	6.9 ± 3.84		13.3 ± 1.2		959 ± 140	
**PMF**	**JAKK2V617**	8 (53.3%)	5/3	56	6/2	12.4 ± 6.9	P=0.1	9.8 ± 3.2	P=0.3	306 ± 227.5	P=0.2
	**JAK2 Wild-type**	7 (46.6%)	3/4	52	4/3	5.9 ± 2.05		7.2 ± 2.1		180 ± 125.8	

**Table 3:** frequency of the JAK2 mutation in other reports

**Country**	**PV (%)**	**ET (%)**	**Method of mutation detection**
Indian, Sazawal S (22)	70%	82%	Polymerase chain reaction and restriction enzyme based assay.
Brazil, Silva R (23)	88%	47%	polymerase chain reaction–restriction fragment length polymorphism
Taiwan, Ho CL (24)	76.2%	46.9%	melting curve analysis
Turkey, Karkucak M (25)	80 %	42 %	tetra-primer polymerase chain reaction
Malaysia, Hamidah NH (26)	95.8%	52.9%	allele specific PCR, ARMS-PCR and RQ-PCR
Lebanon, Mahfouz RA (27)	100%	68.29%	Real-time polymerase chain reaction
China, Chao HY (28)	97%	59.6%	ARMS-PCR, capillary electrophoresis

Also, treated and untreated ET patients with the JAK2V617F mutation had higher leukocyte count and hemoglobin concentration. These results are in accordance with the published literature. ^5^^,^^33^  On the other hand, the present study indicated that PV patients at the time of diagnosis carried the highest mean JAK2 V617F allele burden. The allele burden in our cohort, especially in PV patients at the time of diagnosis, was higher than most previously published data.^34^^-^^36^ In fact, our result in PV patients is consistent with a recent report by Edahiro et al.^   5 ^

However, in PMF cohort, allele burden was lower than those observed in other reports  ^5^^,^^6^^,^^37^  that can be due to the low number of patients. In addition, our PMF patients were under treatment. Previous reports have frequently correlated JAK2 V617F allele burden with elevated hematocrit, leukocyte count and high complication rate in MPNs.^38^^-^^42^ Our results indicated no significant correlation between the allele burden and hemoglobin, leukocyte or platelet count for PV and PMF patients in agreement with some reports ^8^^,^^33^  but in ET group, the relationship between the JAK2 V617F allele burden and leukocyte count was significant. Variable results have been obtained in studies of the effects of HU on the mutant allele in patients with MPNs.^12^^-^^15^ In a retrospective study by Girodon et al.^43^ the levels of V617 allele were compared between a cohort of newly-diagnosed PV or ET (48 PV and 50 ET) and patients who had been under HU therapy (15 PV and 25 ET). They found that treated PV patients had a lower % JAK2V617F than PV patients at the time of diagnosis (44% vs. 54%, p=0.02), but there was no difference in % JAK2V617F between the group of ET patients under treatment and those tested at time of diagnosis. Although HU therapy did not modify the V617F allele burden in ET patients (n=9) analyzed by Hussein et al.^   44 ^, Ricksten et al.^   13 ^ reported significantly lower median JAK2V617F levels in both PV and ET patients after initiation of HU therapy compared to the JAK2V617F levels at diagnosis (19.0%, p=0.002 and 4.3%, p=0.012, respectively). Zalcberg et al.^   17 ^ also reported that HU dose impacts hematologic parameters in PV and ET but does not appreciably affect the JAK2V617F allele burden. Our results indicated a significant decrease of allele burden in PV patients who were receiving HU (43% vs. 72%, p=0.005). In contrast, the effect of the myelo suppressive therapy upon the JAK2V617F allele burden was not significant in ET patients under treatment compared to those tested at the time of diagnosis (40% vs. 49%, p=0.1). This finding is comparable with Girodon et al. results.^43^ Our results also revealed older age, higher hemoglobin and lower platelet count in patients with ≥50% mutational load which is consistent with other reports.^39^^-^^40^ In one study,^32^ significant association was demonstrated between the presence of the mutant allele and female gender in patients with PV (83% vs. 64%). In addition, Stein et al.^45^ found a significantly lower allele burden in women with PV than in men. Larsen et al.^46^ also found a higher allele burden in males than in females. In the current study, we could not demonstrate any correlation between jak2 allele burden and gender.

## CONCLUSION

 In summary, this is the first study analyzing the JAK2V617F allele burden in Iranians subjects, and our results revealed that the allele burden of the JAK2 V617F mutation differs among the subtypes of MPNs, similar to Western patients. Our results also showed that the median allele burden of JAK2 V617F was higher in PV. Although we observed similarities in terms of epidemiological parameters associated with the JAK2V617F allele burden between our cohort and others, we found a lower JAK2V617F allele burden in PMF patients. Thus, for PMF patients, larger-scale studies are needed. In addition, our results reinforce the idea that JAK2V617F allele burden are impacted by HU, as evidenced by the decrease in PV group under HU therapy. 

## Custom

Shirin Ferdowsi and Seyed H. Ghaffari are equally contributed to this work.

## References

[1] Tefferi A (2010). Novel mutations and their functional and clinical relevance in myeloproliferative neoplasms: JAK2, MPL, TET2, ASXL1, CBL, IDH and IKZF1. Leukemia.

[2] Vainchenker W, Delhommeau F, Constantinescu SN (2011). New mutations and pathogenesis of myeloproliferative neoplasms. Blood.

[3] Vannucchi AM, Guglielmelli P, Tefferi A (2009). Advances in understanding and management of myeloproliferative neoplasms. CA Cancer J Clin.

[4] Rumi E, Pietra D, Ferretti V (2014). JAK2 or CALR mutation status defines subtypes of essential thrombocythemia with substantially different clinical course and outcomes. Blood.

[5] Edahiro Y, Morishita S, Takahashi K (2014). JAK2V617F mutation status and allele burden in classical Ph-negative myeloproliferative neoplasms in Japan. Int J Hematol.

[6] Kim HR, Choi HJ, Kim YK (2013). Allelic expression imbalance of JAK2 V617F mutation in BCR-ABL negative myeloproliferative neoplasms. PloS One.

[7] Kuriakose E, Vandris K, Wang YL (2012). Decrease in JAK2V617F allele burden is not a prerequisite to clinical response in patients with polycythemia vera. Haematologica.

[8] Ha JS, Kim YK, Jung SI (2012 ). Correlations between Janus kinase 2 V617F allele burdens and clinicohematologic parameters in myeloproliferative neoplasms. Ann Lab Med.

[9] Zhou J, Ye Y, Zeng S (2013). Impact of JAK2 V617F Mutation on Hemogram Variation in Patients with Non-Reactive Elevated Platelet Counts. PloS One.

[10] Park SH, Chi HS, Cho YU (2013). The allele burden of JAK2 V617F can aid in differential diagnosis of Philadelphia Chromosome-Negative Myeloproliferative Neoplasm. Blood Res.

[11] Barbui T (2011). First-Line Therapy and Special Issues Management in Polycythemia Vera and Essential Thrombocythemia.

[12] Besses C, Álvarez‐Larrán A, Martínez‐Avilés L (2011). Modulation of JAK2 V617F allele burden dynamics by hydroxycarbamide in polycythaemia vera and essential thrombocythaemia patients. Br J Haematol.

[13] Ricksten A, Palmqvist L, Johansson P (2008). Rapid decline of JAK2V617F levels during hydroxyurea treatment in patients with polycythemia vera and essential thrombocythemia. Haematologica.

[14] Larsen TS, Pallisgaard N, de Stricker K (2009). Limited efficacy of hydroxyurea in lowering of the JAK2 V617F allele burden. Hematology.

[15] Spanoudakis E, Bazdiara I, Kotsianidis I (2009). Hydroxyurea (HU) is effective in reducing JAK2V617F mutated clone size in the peripheral blood of essential thrombocythemia (ET) and polycythemia vera (PV) patients. Ann Hematol.

[16] Antonioli E, Carobbio A, Pieri L (2010 ). Hydroxyurea does not appreciably reduce JAK2 V617F allele burden in patients with polycythemia vera or essential thrombocythemia. Haematologica.

[17] Zalcberg IR, Ayres-Silva J, de Azevedo AM (2011). Hydroxyurea dose impacts hematologic parameters in polycythemia vera and essential thrombocythemia but does not appreciably affect JAK2-V617F allele burden. Haematologica.

[18] Michiels JJ, Juvonen E, editors Proposal for revised diagnostic criteria of essential thrombocythemia and polycythemia vera by the Thrombocythemia Vera Study Group. Semin Thromb Hemost.

[19] Jones AV, Kreil S, Zoi K (2005). Widespread occurrence of the JAK2 V617F mutation in chronic myeloproliferative disorders. Blood.

[20] Fantasia F, Di Capua EN, Cenfra N (2014). A highly specific q-RT-PCR assay to address the relevance of the JAK2WT and JAK2V617F expression levels and control genes in Ph-negative myeloproliferative neoplasms. Ann Hematol.

[21] Vannucchi A, Pancrazzi A, Bogani C (2006). A quantitative assay for JAK2V617F mutation in myeloproliferative disorders by ARMS-PCR and capillary electrophoresis. Leukemia.

[22] Sazawal S, Bajaj J, Chikkara S (2010). Prevalence of JAK2 V617F mutation in Indian patients with chronic myeloproliferative disorders. Indian J Med Res.

[23] da Silva RR, Domingues HB, Machado C (2012). JAK2 V617F mutation prevalence in myeloproliferative neoplasms in Pernambuco, Brazil. Genet Test Mol Biomarkers.

[24] Ho C-L, Wu Y-Y, Hung H-M (2012). Rapid identification of heterozygous or homozygous JAK2 V617F mutations in myeloproliferative neoplasms using melting curve analysis. Journal of the Formosan Medical Association.

[25] Karkucak M, Yakut T, Ozkocaman V (2012). Evaluation of the JAK2-V617F gene mutation in Turkish patients with essential thrombocythemia and polycythemia vera. Mol Biol Rep.

[26] Hamidah N, Farisah N, Azlinda A (2011). A study of JAK2 (V617F) gene mutation in patients with chronic myeloproliferative disorders. La Clinica Terapeutica.

[27] Mahfouz RA, Hoteit R, Salem Z (2011). JAK2 V617F gene mutation in the laboratory work-up of myeloproliferative disorders: experience of a major referral center in Lebanon. Genet Test Mol Biomarkers.

[28] Chao H, Shen Y, Zhang R (2009). A quantitative assay for JAK2 mutation in 135 patients with chronic myeloproliferative neoplasms. Zhonghua xue ye xue za zhi= Zhonghua xueyexue zazhi.

[29] Baxter EJ, Scott LM, Campbell PJ (2005). Acquired mutation of the tyrosine kinase JAK2 in human myeloproliferative disorders. Lancet.

[30] Zhao R, Xing S, Li Z (2005). Identification of an acquired JAK2 mutation in polycythemia vera. J Biol Chem.

[31] James C, Ugo V, Le Couédic JP (2005). A unique clonal JAK2 mutation leading to constitutive signalling causes polycythaemia vera. Nature.

[32] Levine RL, Wadleigh M, Cools J (2005). Activating mutation in the tyrosine kinase JAK2 in polycythemia vera, essential thrombocythemia, and myeloid metaplasia with myelofibrosis. Cancer cell.

[33] Antonioli E, Guglielmelli P, Poli G (2008). Influence of JAK2V617F allele burden on phenotype in essential thrombocythemia. Haematologica.

[34] Vannucchi A, Antonioli E, Guglielmelli P (2008). Clinical correlates of JAK2V617F presence or allele burden in myeloproliferative neoplasms: a critical reappraisal. Leukemia.

[35] Vannucchi A, Antonioli E, Guglielmelli P (2007). Prospective identification of high-risk polycythemia vera patients based on JAK2V617F allele burden. Leukemia.

[36] Kittur J, Knudson RA, Lasho TL (2007). Clinical correlates of JAK2V617F allele burden in essential thrombocythemia. Cancer.

[37] Alshemmari SH, Rajaan R, Ameen R (2014). JAK2V617F allele burden in patients with myeloproliferative neoplasms. Ann Hematol.

[38] Silver RT, Vandris K, Wang YL (2011). JAK2 V617F allele burden in polycythemia vera correlates with grade of myelofibrosis, but is not substantially affected by therapy. Leuk Res.

[39] Tefferi A, Lasho TL, Schwager SM (2006). The clinical phenotype of wild‐type, heterozygous, and homozygous JAK2V617F in polycythemia vera. Cancer.

[40] Vannucchi AM, Antonioli E, Guglielmelli P (2007). Clinical profile of homozygous JAK2 617V> F mutation in patients with polycythemia vera or essential thrombocythemia. Blood.

[41] Passamonti F, Rumi E, Pietra D (2010). A prospective study of 338 patients with polycythemia vera: the impact of JAK2 (V617F) allele burden and leukocytosis on fibrotic or leukemic disease transformation and vascular complications. Leukemia.

[42] Tefferi A, Strand J, Lasho T (2007). Bone marrow JAK2V617F allele burden and clinical correlates in polycythemia vera. Leukemia.

[43] Girodon F, Schaeffer C, Cleyrat C (2008). Frequent reduction or absence of detection of the JAK2-mutated clone in JAK2V617F-positive patients within the first years of hydroxyurea therapy. Haematologica.

[44] Hussein K, Bock O, Theophile K (2009). JAK2( V617F) allele burden discriminates essential thrombocythemia from a subset of prefibrotic-stage primary myelofibrosis. Exp Hematol.

[45] Stein BL, Williams DM, Wang NY (2010). Sex differences in the JAK2V617F allele burden in chronic myeloproliferative disorders. Haematologica.

[46] Larsen TS, Pallisgaard N, Møller MB (2007). The JAK2 V617F allele burden in essential thrombocythemia, polycythemia vera and primary myelofibrosis–impact on disease phenotype. Eur J Haematol.

